# Modulating Tumour Hypoxia in Prostate Cancer Through Exercise: The Impact of Redox Signalling on Radiosensitivity

**DOI:** 10.1186/s40798-022-00436-9

**Published:** 2022-04-08

**Authors:** Malcolm Brown, Amélie Rébillard, Nicolas H. Hart, Dominic O’Connor, Gillian Prue, Joe M. O’Sullivan, Suneil Jain

**Affiliations:** 1 School of Nursing and Midwifery, Queen’s University Belfast, Northern Ireland Belfast, UK; 2 Movement, Sport and Health Sciences Laboratory, Université Rennes 2, ENS Rennes, Bruz, France; 3 College of Nursing and Health Sciences, Flinders University, Adelaide, SA Australia; 4 School of Medical and Health Sciences, Edith Cowan University, Perth, WA Australia; 5 Institute for Health Research, University of Notre Dame Australia, Perth, WA Australia; 6 School of Health Sciences, University of Nottingham, Nottingham, England, UK; 7 The Patrick G Johnston Centre for Cancer Research, Queen’s University Belfast, Belfast, Northern Ireland UK

**Keywords:** Exercise, Prostate cancer, Hypoxia, Radiotherapy, Skeletal muscle, Reactive oxygen species

## Abstract

Prostate cancer is a complex disease affecting millions of men globally. Radiotherapy (RT) is a common treatment modality although treatment efficacy is dependent upon several features within the tumour microenvironment (TME), especially hypoxia. A hypoxic TME heightens radioresistance and thus disease recurrence and treatment failure continues to pose important challenges. However, the TME evolves under the influence of factors in systemic circulation and cellular crosstalk, underscoring its potential to be acutely and therapeutically modified. Early preclinical evidence suggests exercise may affect tumour growth and some of the benefits drawn, could act to radiosensitise tumours to treatment. Intracellular perturbations in skeletal muscle reactive oxygen species (ROS) stimulate the production of numerous factors that can exert autocrine, paracrine, and endocrine effects on the prostate. However, findings supporting this notion are limited and the associated mechanisms are poorly understood. In light of this preclinical evidence, we propose systemic changes in redox signalling with exercise activate redox-sensitive factors within the TME and improve tumour hypoxia and treatment outcomes, when combined with RT. To this end, we suggest a connection between exercise, ROS and tumour growth kinetics, highlighting the potential of exercise to sensitise tumour cells to RT, and improve treatment efficacy.

## Key Points

- A lack of oxygen to the tumour (hypoxia) impairs radiotherapy treatment efficacy.
- Exercise may beneficially alter components of the TME and act as a sensitiser for treatment.
- Reactive oxygen species generated by exercise and redox-sensitive mechanisms within the tumour, alongside a host of other exercise-induced mechanisms, may prove pivotal in facilitating the treatment effects.

## Introduction

Prostate cancer (PCa) is the most diagnosed male malignancy in the developed world, with an annual incidence of approximately 1.3 million cases [1]. Presently, an estimated 400,000 men in the United Kingdom are living with and beyond PCa, many of whom will have received radiotherapy (RT) as part of their treatment. Principally, RT irreversibly damages tumour cell DNA via ionisation and generation of reactive oxygen species (ROS), inducing cell death. Despite significant advances to enhance treatment efficacy, disease recurrence continues to pose challenges, with treatment resistance one of the main causes of treatment failure [2]. Indeed, radioresistance and recurrence are mediated by complex, multifocal processes that occur in a highly plastic tumour microenvironment (TME), with hypoxia recognised as a major obstacle in treatment response and linked to poorer prognoses [3–5]. Prostate tumours are markedly hypoxic, stimulating conditions that regulate the multistep progression of cancer [6–9]. However, the TME may prove a legitimate therapeutic target as it constantly evolves under the influence of systemic milieu and cellular crosstalk in the surrounding stroma. Novel strategies that selectively sensitise prostate tumours to treatment methods are urgently required.

Chemical radiosensitisers have proven to be effective complementary agents for RT treatment [10, 11], although there is still no accepted standard of care for alleviating tumour hypoxia [12]. Findings from exercise trials are promising but conclusive evidence remains absent. To date, exercise has proven to be a safe, feasible, and effective adjuvant therapy and many of the beneficial responses invoked could prove to be natural radiosensitisers (e.g. increased tumour blood flow [13]). However, the biological mechanisms remain speculative and we propose redox signalling as a plausible stimulus underpinning improved tumour hypoxia. Exercise provokes a transient disruption in homeostasis, leading to the generation of ROS, from several cellular sources [14]. Whilst historically viewed as detrimental, driving oxidative stress and numerous chronic conditions, physiological concentrations of ROS are emerging as integral regulators of cellular adaptation and subcellular messengers in signal transduction, driving the widespread benefits of exercise [14–16]. The paradoxical, bi-functional nature of ROS, particularly the redox signalling perspective, may go some way to explaining how exercise-induced factors may positively impact prostate tumours. A recent review suggests that muscle-derived cytokines, termed ‘myokines’, have the capacity to favourably manipulate tumour environmental conditions, following their secretion into systemic circulation [17]. Mechanistically, the molecular mechanisms remain poorly understood but myokine kinetics appear redox-sensitive, involving the induction of p38 mitogen-activated protein kinase (MAPK) and putative transcriptional regulation of nuclear factor kappa-light-chain-enhancer of activated B cells (NF-κB) [18]. While conclusive evidence is limited, several reviews commonly support this mechanism, given their mutual signalling pathways, and ROS thus could conceivably influence a parallel rise in cytokine secretion. We suggest that adaptations within the tumour may occur, downstream of redox-sensitive mechanisms commencing within skeletal muscle, and that the collective and widespread response to exercise can act as a viable radiosensitiser. Thus, exercise may affect redox signalling within the tumour, improving hypoxia and PCa treatment outcomes, when combined with RT.

## Neovascularisation and Cycling Hypoxia: Drivers of Treatment Resistance?

Malignant tumours stimulate neovascularisation to sustain growth. Normally, angiogenesis is a highly regulated process; however inhospitable conditions within the TME and accumulating growth factors trigger an ‘angiogenic switch’, whereby the natural balance between proangiogenic and antiangiogenic factors is disturbed [19, 20]. This phenotypical switch is promoted by vascular endothelial growth factor (VEGF) and complementary growth factors [21]. Tumour vascularisation is therefore a multistep process culminating in a network of blood vessels that are dysfunctional [22]. Characteristically, tumour blood vessels are disorganised, tortuous, and immature, leading to increased interstitial pressure, impaired perfusion, and hypoxic regions. Cycling (intermittent) hypoxia relates to temporally unstable oxygen transport, promoting transient fluctuations in tumour perfusion and hypoxia [23]. This hypoxia-reoxygenation pattern affects cells immediately adjacent to inefficiently perfused vessels, with reperfusion generating ROS [24]. This collection of processes enables hypoxia-inducible factor 1α (HIF-1α) stabilisation and the co-activation of NF-κB, which complementarily mediate the expression of genes controlling anaerobic metabolism, angiogenesis, treatment resistance and metastases [25, 26].

Cells exposed to cycling hypoxia exhibit a robust stabilisation and accumulation of HIF-1α, leading to a more aggressive phenotype and reduced responsiveness to RT [24, 27]. Moeller and colleagues [28] reported that following exposure to ionising radiation, reoxygenation significantly increased ROS, accompanied by the stabilisation of HIF-1α and expression of HIF-1α-dependent genes. This was accompanied by increased VEGF expression (mediated by HIF-1α), conferring heightened radioresistance. Given that the activation of HIF-1α and NF-κB during cycling hypoxia is partly mediated by increased ROS production, this further implicates ROS in cancer progression [26]. ROS are generated during reoxygenation (up to 100-fold from basal levels) via several cellular sources (e.g. tumour cell mitochondria; NADPH oxidase; xanthine oxidase) triggering an increase in metabolic activity and tumour growth [24]. In non-malignant cells, intracellular antioxidant enzymes and non-enzymatic antioxidants detoxify ROS, but within the TME, sustained, supraphysiological concentrations of ROS overwhelm coping mechanisms and a state of oxidative stress ensues [29]. The aberrant generation of ROS and the inherent decrease in antioxidant capacity contribute to greater oxidative damage and stimulate several metabolic sensors, transcription factors and genes that permit cancer progression [6]. While ROS are associated with harmful biological events in this context, lower concentrations are essential signalling molecules for health [30]. In particular, ROS generated during exercise act as important subcellular messengers, stimulating several kinases involved in gene expression and cellular adaptation [31].

## The Potential Impact of Exercise on Tumour Growth?

A recent meta-analysis confirmed higher levels of post-diagnosis physical activity can reduce PCa-specific mortality [32]. This important finding reflects the mounting observational evidence suggesting exercise training can reduce the rate of prostate tumour progression [33]. Greater insights can be gathered from preclinical trials [34–39]. Rundqvist and colleagues [40] harvested LNCaP cells with exercise serum (drawn after 60 min of incremental cycling exercise) and reported a growth inhibitory effect (31%), which persisted 96 h post-exercise. Similarly, exercise training in rats has been reported to reduce cancer cell viability in serum and the prostate [41]. Although neither trial examined the tumour vasculature, it is conceivable that improved tumour blood flow may contribute to such a response. Preclinical trials have shown that exercise acutely increases tumour perfusion (twofold) and concomitantly decreases hypoxia [13, 42–45]. Consistent with physiological adaptation, transient increases in perfusion accumulate over time, stimulating vascularisation, and decreasing the trajectory of local tumours and propensity for metastases [46]. Exercise has also been purported to assist in remodelling tumour vasculature, thus delaying tumour growth in patients with pancreatic ductal adenocarcinoma [47].

**Fig. 1:**
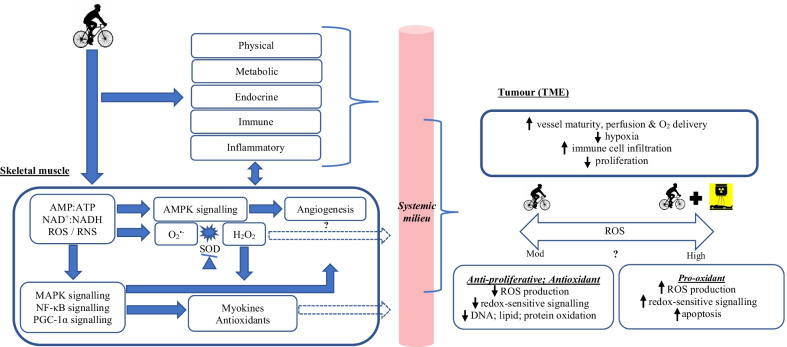
Simplified, hypothetical mechanism(s) whereby exercise may modulate aspects of the TME and potential antioxidant / pro-oxidant responses**.** Exercise stimulates numerous responses that influence the systemic milieu and may modify components of the TME. Naturally, the onset of exercise immediately promotes a cardiorespiratory response, potentially enhancing tumour perfusion and O_2_ delivery. Within skeletal muscle, enhanced metabolic demand and the generation of ROS activate several intracellular signalling pathways and key transcriptional factors, culminating in the expression of genes encoding antioxidant enzymes and myokines (and potentially other relevant factors). Theoretically, exercise-induced reactive derivatives and/or the subsequent expression of myokines into circulation (dashed arrows), may influence the systemic milieu and hypoxic signalling, driving anti-proliferative, antioxidant signalling intrinsically. Alternatively, vigorous exercise alongside radiotherapy could conceivably create sufficiently high (toxic) concentrations within the TME, inducing apoptosis (both potential theories marked with a question mark as presently unknown). The potential role of H_2_O_2_ remains poorly understood (unknown signalling properties and membrane permeability in this model marked with a question mark), though it may serve as an important signalling factor. *Abbreviations* AMP, adenosine monophosphate; AMPK, AMP-activated protein kinase; ATP, adenosine triphosphate; DNA, deoxyribonucleic acid; H_2_O_2_, hydrogen peroxide; MAPK, mitogen-activated protein kinase; Mod, moderate; NAD, nicotinamide adenine dinucleotide; NF-κB, nuclear factor kappa-light-chain-enhancer of activated B cells; O_2_, oxygen; O_2_^•−^, superoxide; PGC-1α, peroxisome proliferator-activated receptor-gamma coactivator; RNS, reactive nitrogen species; ROS, reactive oxygen species; SOD, superoxide dismutase; TME, tumour microenvironment

Several reviews posit exercise as an anti-tumour therapy, alongside contemporary treatment methods, through regulating the systemic milieu and conceivably tumour intrinsic factors [3, 48–51]. An initial study examining aerobic exercise alongside neoadjuvant chemotherapy, reported that 12-weeks of cycling can modulate several host and tumour related pathways during treatment [52]. Exercise acutely triggers a systemic response integrative of multiple organs. Skeletal muscle is primarily solicited during exercise, with intracellular perturbations in ROS stimulating numerous metabolic sensors (e.g. AMP-activated protein kinase), enhancing the expression of several genes [53] (Fig. 1). The production of such factors can exert autocrine, paracrine, and endocrine effects on several neighbouring and distant tissues, including prostate tumour cells [49, 53]. A recent review speculates this crosstalk may be stimulated by spikes in ROS, in a wave-like fashion, that mediate several redox-sensitive mechanisms to propagate remote signalling [54]. Thus, potential intratumoural adaptations may be a multistep process commencing in, or driven by skeletal muscle [53] (Fig. 1). However, at this time there is a dearth of evidence delving into the potential intratumoural, anti-cancer mechanisms and outcomes in humans, though it appears frequently engaging in vigorous physical activity normalises the prostate tumour vasculature [55], improves oxygenation, and ultimately cancer prognosis [23].

## Could Exercise Radiosensitise Prostate Tumours?

Given the potential of exercise to influence tumour vasculature, oxygenated blood perfusion, and thus hypoxia, it follows that exercise could act as a radiosensitiser, reducing radioresistance. Treatment response relies on well-oxygenated tissue (permitting oxygen fixation) and at lower partial pressures of oxygen, radiosensitivity is considerably reduced [56]. Preclinical research suggests exercise training has the capacity to restore oxygen delivery and potentially modulate the TME, providing a theoretical foundation to enhance radiotherapeutic potency [13, 42–45]. Exercise training may also mediate several signalling pathways that sensitise the tumour to treatment. It is currently unproven whether exercise benefits RT or whether exercise-induced redox signalling holds any definitive therapeutic effects. However, a recent in vivo trial reported RT combined with exercise training reduced prostate tumour oxidative damage and slowed tumour growth compared to RT alone [57]. Indeed, this finding is supported by pending preclinical data suggesting exercise improves the tumour response to RT as a result of reduced tumour hypoxia [48]. Moreover, this study also reported exercise reduced oxidative damage to DNA in mice (4T1 tumour sections stained for 8-oxo-dG) indicating modified redox signalling within the TME [48]. Taken together, this suggests a link between exercise, ROS and tumour growth kinetics and highlights the potential of exercise to sensitise tumour cells to RT, and improve treatment efficacy (Fig. 1). Further, exercise training has the capacity to slow the rate of progression, reduce the accumulation of myeloid-derived suppressor cells (MDSCs) and restore tumour immunity (e.g. increased NK-cells) in a mouse model of 4T1 mammary carcinoma [58]. ROS regulate MDSCs and MDSCs reciprocally use ROS to fulfil their immunosuppressive actions [59], suggesting yet again a potentially mediatory role. This collective response to exercise improved the efficacy of immunotherapy and RT treatment [58], raising the notion that exercise may invoke a similar response in the prostatic model. Thus, exercise on one hand could stimulate anti-proliferative, antioxidant effects in accordance with the theory of ‘*hormesis*’ [60] or on the other hand, prooxidant apoptosis in synergy with standard therapies, in line with oxidative damage [53]. Despite these positive preclinical findings, definitive evidence on the role of ROS and / or redox-sensitive signalling on tumour hypoxia in humans remains unknown and underscores a necessity for further research.

## Discussion

Preclinical findings concerning exercise and the TME have provoked considerable research and clinical interest pertaining to the mechanisms of action surrounding tumour hypoxia and RT efficacy. The apparent intensity-dependent nature of the responses (METs ≥ 6-h week^−1^) and seemingly protective effects [33, 61, 62] are striking and lead us to propose an important mediatory role for redox signalling in this process (alongside numerous other beneficial responses). At a molecular level within skeletal muscle, redox-sensitive mechanisms are stimulated in response to exercise of sufficient intensity and can modulate systemic inflammation (e.g. through the secretion of myokines), metabolism, hormones, and angiogenesis. We believe exercise has the capacity to partly modulate the prostate TME via alterations in the systemic milieu and propose that exercise-induced redox signalling plays a crucial mediatory role in these beneficial responses (Fig. 1). Given ROS indirectly influence the activity of several gene families, transcription factors and modify integral molecular components, they could conceivably reprogramme the TME with exercise training. Specifically, exercise-induced redox signalling indirectly modulates VEGF, HIF and NF-κB signalling processes [63], potentially reducing the expression of prometastatic genes, while increasing protein kinase signalling activity (similarly to skeletal muscle); ultimately improving the profile of host anti-cancer factors in systemic circulation and reducing the likelihood of an aggressive phenotype [52].

At this time, data supporting this mechanism are limited to a handful of studies. In addition to those mentioned in the preceding sections, Repka and Hayward [64] reported exercise training reduced blood DNA-damage and protein oxidation, while increasing systemic antioxidant capacity. Whether systemic changes in oxidative parameters extend to the tumour or exert mediatory effects on the TME is inconclusive; however, forced treadmill exercise training was shown to reduce prostate tumour proliferation in rats bearing AT-1 tumours, a change attributed to reduced tumour DNA oxidation and lipid peroxidation [36]. The extent of this decrease in tumour oxidative stress could be up to threefold in exercising patients [48] with antioxidant supplementation blunting this response, highlighting the importance of redox-sensitive signalling in intratumoural adaptation. The source of the intrinsic changes in oxidative damage remains unconfirmed, but may be a consequence of the systemic milieu or secondary local alterations in the TME. Conceivably, systemic changes in redox signalling with exercise, could activate redox-sensitive antioxidant genes within the tumour (possibly through kelch-like ECH-associated protein 1-nuclear factor (erythroid-derived 2)-like 2 (Keap-Nrf2) and PGC-1α) to attenuate oxidative damage [53].

## Conclusion

While the effects of exercise on PCa treatment-related side effects are reasonably established, the influence of exercise on RT and the reputed mechanisms of action, require further investigation. Further enquiry will assist in identifying a minimum effective / optimal dose, appropriate modalities of exercise, and a time course for observed responses, particularly relating to RT regimens. Distinguishing whether the acute effects of exercise (immediately prior to treatment) or gradual, chronic effects sensitise tumour tissue for RT would be highly advantageous, further to whether these mechanisms amplify treatment efficacy and tolerance. Exercise training may in some part, reprogramme the systemic milieu towards an anticancer phenotype, reducing tumour hypoxia and tumour proliferation through biological, epigenetic, metabolic, and inflammatory mechanisms. Redox signalling is integral in this beneficial response to exercise, though limited quantitative data exist pertaining to the influence of such species (modest and high levels) within the TME and their effect on human cancer cells [65].

## Data Availability

Not applicable.
